# A novel application of capnography during controlled human exposure to air pollution

**DOI:** 10.1186/1475-925X-5-54

**Published:** 2006-10-18

**Authors:** Karl Z Lukic, Bruce Urch, Michael Fila, Marie E Faughnan, Frances Silverman

**Affiliations:** 1 Gage Occupational and Environmental Health Unit, St. Michael's Hospital & University of Toronto, Toronto, ON, Canada; 2 Institute of Medical Sciences, University of Toronto, Toronto, ON, Canada; 3 Department of Public Health Sciences, University of Toronto, Toronto, ON, Canada; 4 Department of Medicine, University of Toronto, Toronto, ON, Canada; 5 Department of Chemical Engineering, University of Toronto, Toronto, ON, Canada; 6 Division of Respiratory Medicine, Department of Medicine, St. Michael's Hospital, Toronto, ON, Canada

## Abstract

**Background:**

The objective was to determine the repeatability and stability of capnography interfaced with human exposure facility.

**Methods:**

Capnographic wave signals were obtained from five healthy volunteers exposed to particle-free, filtered air during two consecutive 5 min intervals, 10 min apart, within the open and then the sealed and operational human exposure facility (HEF). Using a customized setup comprised of the Oridion Microcap^® ^portable capnograph, DA converter and AD card, the signal was acquired and saved as an ASCII file for subsequent processing. The minute ventilation (VE), respiratory rate (RR) and expiratory tidal volume (V_TE_) were recorded before and after capnographic recording and then averaged. Each capnographic tracing was analyzed for acceptable waves. From each recorded interval, 8 to 19 acceptable waves were selected and measured. The following wave parameters were obtained: total length and length of phase II and III, slope of phase II and III, area under the curve and area under phase III. In addition, we recorded signal measures including the mean, standard deviation, mode, minimum, maximum – which equals end-tidal CO_2 _(EtCO_2_), zero-corrected maximum and true RMS.

**Results:**

Statistical analysis using a paired t-test for means showed no statistically significant changes of any wave parameters and wave signal measures, corrected for RR and V_TE_, comparing the measures when the HEF was open vs. sealed and operational. The coefficients of variation of the zero-corrected and uncorrected EtCO_2_, phase II absolute difference, signal mean, standard deviation and RMS were less than 10% despite a sub-atmospheric barometric pressure, and slightly higher temperature and relative humidity within the HEF when operational.

**Conclusion:**

We showed that a customized setup for the acquisition and processing of the capnographic wave signal, interfaced with HEF was stable and repeatable. Thus, we expect that analysis of capnographic waves in controlled human air pollution exposure studies is a feasible tool for characterization of cardio-pulmonary effects of such exposures.

## Background

The primary objective of current and future air pollution studies is to further understand the mechanisms for epidemiologic data on increased morbidity and mortality due to exposure to a ambient pollutants, e.g. fine particulate air pollution, i.e. particles of aerodynamic diameter < 2.5 μm (PM_2.5_) [1-5]. In addition, the next objective is to link the data from animal studies [6] with clinical evidence related to adverse cardio-pulmonary and vascular events; in particular, coronary and cerebro-vascular events related with such exposure [7-10]. To this end, environmental air pollution studies aim to reveal the physiologic changes and molecular mechanisms that follow inhalation of gaseous and particulate pollution and lead to a systemic inflammatory response. Moreover, they aim to provide plausible explanations of the association between air pollution exposure and morbidity/mortality from cardio-pulmonary and vascular etiology. Understanding these outcomes is of paramount importance in susceptible populations such as children, the elderly, asthmatics, patients suffering from chronic obstructive pulmonary disease (COPD), cardiovascular diseases and diabetes.

Different health outcomes and indices have been assessed during controlled human exposures to gaseous and particulate air pollution: ECG and lung function changes [11,12], heart rate variability [13-15], blood and sputum markers of inflammation – cellular variability and mediators including interleukin 6 (IL-6), leukotriene B4 (LTB_4_), tumor necrosis factor alpha (TNFα,) interleukin 8 (IL-8), endothelins and fibrinogen, [13,16].

Acute PM_2.5 _exposure can promote systemic inflammation by activating the capillary endothelium in the pulmonary circulation [17,18]. Therefore, we redirected our interests in detecting acute effects of PM_2.5 _exposure from lung mechanics to gas exchange surface of the alveolar-capillary membrane. Our fine concentrated ambient particles (CAP) exposure facility can concentrate particles sized 0.15 – 2.5 μm in aerodynamic diameter. Due to geometry of the airways, fine particles (PM_2.5_) can be inhaled deep into the small conducting bronchioles and the alveoli. If adverse effects of PM_2.5 _are to be measured, a major site that could be examined is the alveolar-capillary membrane. Therefore, we considered capnography as the most suitable procedure that can provide real-time information about alveolar ventilation, pulmonary perfusion, respiratory pattern, and CO_2 _elimination/production. Clinically, capnography has been used in concert with pulse oxymetry to detect adverse respiratory events during anesthesia [19,20]. In addition, capnography has been used in emergency rooms and during emergency transportation [21-24]. However, although widely used in clinical and pre-hospital settings, an extensive literature search confirmed that capnography has not been used as a procedure or outcome measure in settings of controlled human air pollution studies.

In our previous studies [17,25], we could not detect any statistically significant changes in standard pulmonary function measures, such as forced expiratory volume in the 1^st ^second (FEV_1_) or lung diffusing capacity for carbon monoxide (D_L_CO). However, we have reported a constriction of the brachial artery immediately following a 2-hour CAP + O_3 _exposure [18] and an acute increase in diastolic blood pressure in healthy adults during controlled exposure to CAP ± O_3 _[26]. We hypothesize that "real-time" capnography would be a more sensitive or even specific marker of the inflammation induced by pollutants (CAP ± O_3_) at the alveolar-capillary membrane, as well as pollutant-induced airway constriction, rather than more gross, effort dependent functional tests such as spirometry and lung diffusion. Since events at the alveolar-capillary membrane are an instantaneous reflection of ventilatory/cardiovascular function, any compromise of the ventilation-perfusion relationship, which may result after breathing CAP ± O_3_, could have some detectable consequences. Therefore, capnography as a non-invasive, instantaneous physiological measurement, which could provide insight into alveolar ventilation, physiological dead space changes, pulmonary perfusion, ventilation-perfusion mismatch, changes of cardiac output and respiratory patterns, seems to be a potentially valuable and needed procedure in air pollution research.

**Figure 1:**
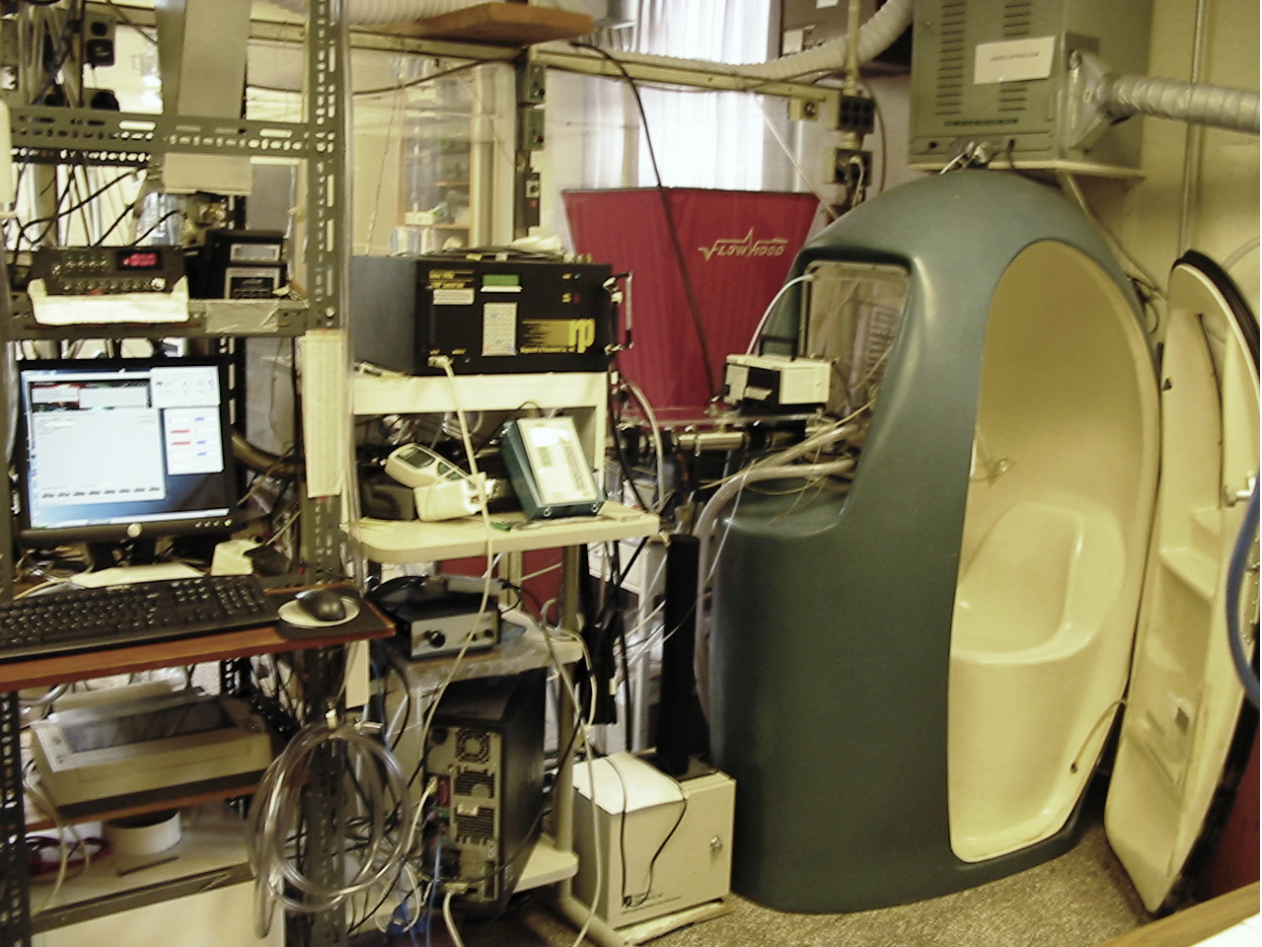
The photo of HEF with door open.

In this study, we tested acquisition of the capnographic wave signal for stability and repeatability through customized hardware & software components interfaced with the human exposure facility of The Gage Occupational and Environmental Health Unit, St. Michael's Hospital and University of Toronto. The HEF design and exposure characterization have been described in details elsewhere [17,25]. See Figure 1 showing HEF with the door open.

## Methods

### Hardware and Software Setup

**Figure 2:**
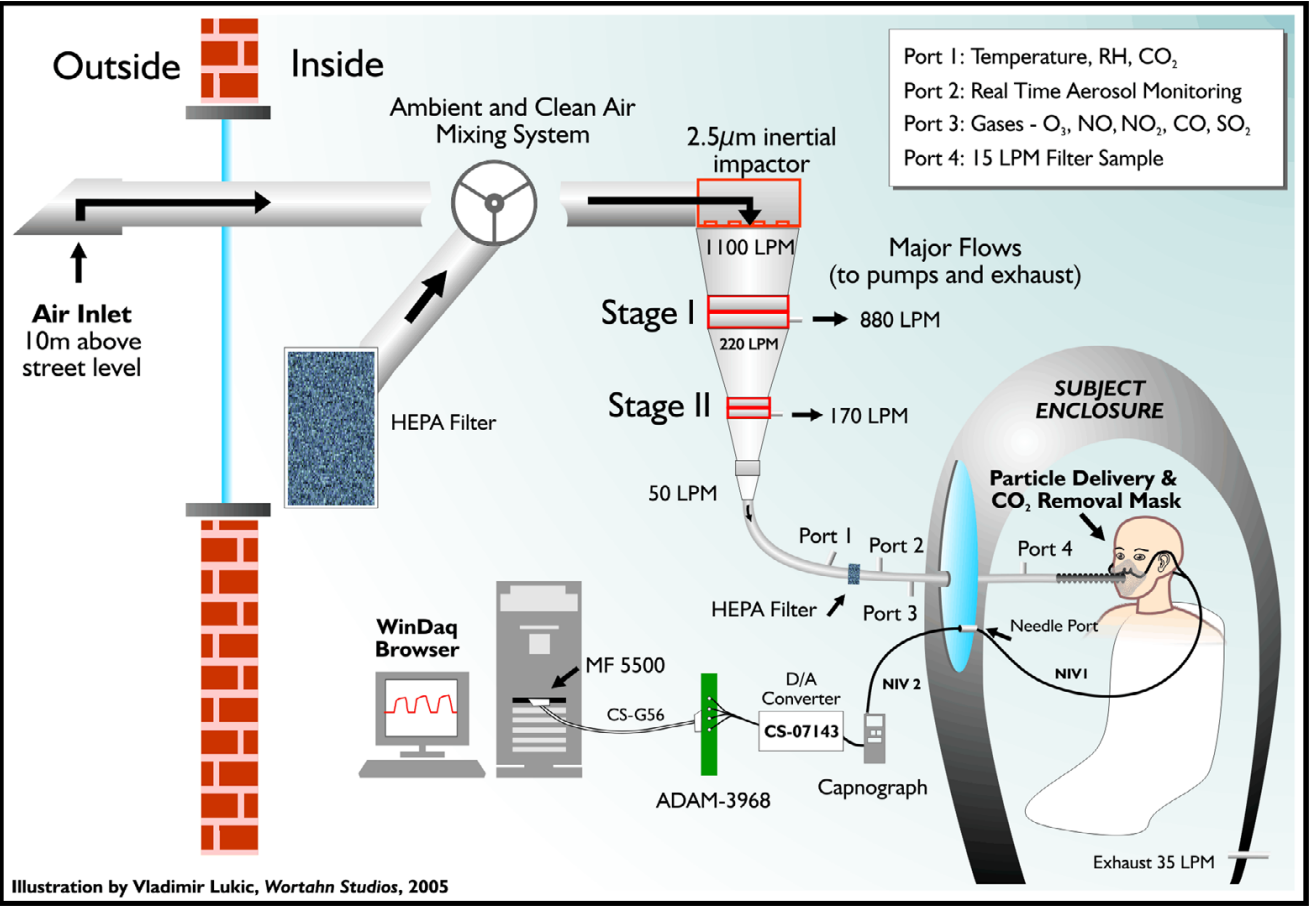
**Capnography components added to existing HEF design**. This illustration presents customized hardware & software components for the capnographic wave signal acquisition, interfaced with existing HEF used for the air pollution studies.

See Figure 2 for the schematic of the hardware interfaced with the HEF, which was used for this procedure. In order to record and analyze instantaneous CO_2 _digitized waveforms, we chose the commercially available Oridion Microcap^® ^CO_2 _portable bedside capnograph (Oridion Systems Ltd, Microcap^®^, model #CS-04178) [27]. It uses Microstream^® ^Technology with low-flow, sidestream aspiration vs. traditional high-flow or mainstream monitors [28,29]. This monitor has a low sampling rate of 50 ml/min and an accuracy of EtCO_2 _readings of ± 2 mmHg in the range of 0–38 mmHg and ± 5% of the reading in the range of 39–99 mmHg, when steady state is reached. EtCO_2 _accuracy is maintained for up to 80 breaths/min. The system response time, including delay and rise time, is typically 2.45 seconds, with a maximum 2.9 seconds. In addition, proprietary Microbeam IR source generates only the specific wavelengths characteristic of the CO_2 _absorption spectrum. This IR source illuminates the microsample cell and reference channel, where the infrared emission exactly matches the absorption spectrum of the CO_2 _molecule, thus other gases in the sample such as N_2_O, O_2 _and water vapor, do not affect the CO_2 _reading. The capnograph has a built-in self-check procedure for verification of validity of the recorded signal. These features make it suitable for our HEF setting, in which we expose non-intubated healthy subjects and subjects with mild pulmonary or cardiovascular disease.

The capnograph we used, had a factory digital-output-only, although, as an option the manufacturer provided a Digital-to-Analog Converter (CS-07143) [30], which was used to process out 4 digital signals including ground, CO_2 _wave, respiratory rate and CO_2 _measurement validity (indicates invalidity raising output voltage to full scale). At full scale, 0.9 V equals 100 mmHg for the CO_2 _output and 150 breaths/min for the respiratory rate output. A voltage of 1.0 V indicates invalidity due to sampling line disconnection, auto zero, and/or malfunction in all channels. An analog output from DA converter was directed to the terminal board ADAM – 3968 SCSI 68P by Super Logics Inc. and further by an expansion cable CS-G56 as a single-ended connection to ADAC MF-5500, 12-bit, 100-kHz PCI data acquisition board [31]. The CO_2 _wave signal resolution obtained through entire setup (capnograph, DA converter and AD card) was 0.27874 mmHg over the full scale of 100 mmHg. The acquired signal was captured by WinView software v. 2.9 from Super Logic Inc [32], using the continuous sampling mode with 20 samples per second, board DMA and smooth scanning mode. Logged data were saved as an ASCII file on the hard drive. The computer used was a Dell Optiplex GX270 with 3.2 GHz Pentium 4 processor and 512 Mb RAM. Saved ASCII files were imported into WinDaq Waveform Browser version 2.39 by DATAQ Instruments Inc. [33], where capnographic waves were analyzed for acceptability according to a Landis score [34]. Variables of interest were measured and entered into an MS Access 2003 database.

The exhaled CO_2 _was sampled using two customized Microstream^® ^EtCO_2 _adult NIV Lines™ [35], joined together. We did not want to place the capnograph itself within the sealed HEF, which is at sub-atmospheric pressure (11 mmHg), and slightly higher temperature and relative humidity compared to laboratory and ambient conditions, respectively. The first line (NIV-1) had the capnograph end cut off and that end was attached inside the HEF to the 20G diameter custom needle port, installed through Plexiglas window. The subject used the patient end as a nasal probe with prongs placed comfortably within the nostrils. The second line (NIV-2) had the patient end cut off and that end was attached to the other side of the needle port. The capnograph end of this line was attached to the sampling line port of the capnograph itself. The total length of sampling line, joined in this fashion, was 4.3 m.

### Subjects

Two tests (EXP#1 and EXP#2) were carried out on five healthy, non-smokers (4 males and 1 female), aged 35–46. Continuous capnographic wave recordings were obtained twice on each subject, 10 min apart, while seated inside the HEF, first with the door open and then with the door sealed under operational conditions. Subjects had the sampling line nasal prongs placed inside their nostrils, with a particle delivery/CO_2 _removal mask over their nose and mouth. Monitoring and routine testing was carried out as in our human exposure studies during a filtered air control exposure [17,25]. One subject had variable and completely unacceptable waveforms due to periodical sniffling during the tests, thus we analyzed data for four subjects. Immediately before and after each capnographic waveform recording, resting expired tidal volume and respiratory rate were measured using a VMM-401 flow turbine by Interface Associates [37]. It was essential to directly measure tidal volume and respiratory rate, as changes in these parameters can modify the EtCO_2 _[38]. The protocol was approved by the Research Ethics Board of St. Michael's Hospital, Toronto.

### Data and Statistical Analysis

**Figure 3:**
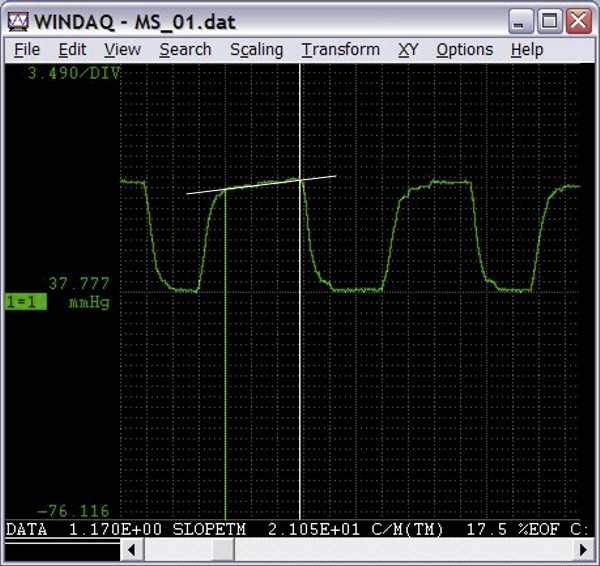
**The example of capnographic wave analysis using WinDaq software**. Screenshot of WinDaq windows showing the measurement of slope3 of the typical capnographic wave.

We measured acceptable capnographic waves for two sets of variables (wave parameters and wave statistics) and two ventilatory variables, shown in Table 1. Selected capnographic wave parameters are described in the literature with defined physiologic meaning and with the ability to characterize certain ventilatory changes [34,39,40]. Capnographic wave signal statistics represented standard statistical parameters, noting that RMS is derived as true RMS by means of an appropriate algorithm within WINDAQ software, and in this case represented mean expired CO_2 _(expressed in mmHg) over the expiratory phase of one breath. The mathematical definition of true RMS used by WINDAQ to derive this statistics is ∑n=1W(νn)2W
 MathType@MTEF@5@5@+=feaafiart1ev1aaatCvAUfKttLearuWrP9MDH5MBPbIqV92AaeXatLxBI9gBaebbnrfifHhDYfgasaacH8akY=wiFfYdH8Gipec8Eeeu0xXdbba9frFj0=OqFfea0dXdd9vqai=hGuQ8kuc9pgc9s8qqaq=dirpe0xb9q8qiLsFr0=vr0=vr0dc8meaabaqaciaacaGaaeqabaqabeGadaaakeaadaGcaaqaamaalaaabaWaaabmaeaadaqadaqaaiabe27aUnaaBaaaleaacqWGUbGBaeqaaaGccaGLOaGaayzkaaWaaWbaaSqabeaacqaIYaGmaaaabaGaemOBa4Maeyypa0JaeGymaedabaGaem4vaCfaniabggHiLdaakeaacqWGxbWvaaaaleqaaaaa@3A9C@ where: v_n _= instantaneous voltage and W = window size [41]. Measurements such as mean blood pressure and/or flow from a pulsatile pressure, airflow waveform and of course capnographic wave are non-sinusoidal waveforms that require a true RMS calculation. Breathing pattern, low respiratory rate and large tidal volume, can influence the shape of the capnographic wave, particularly the slope of phase III of the capnographic wave [42]; therefore, we recorded these parameters for the purpose of the quality control of the recorded capnographic wave parameters and statistics during the two tests. See Figure 3 showing the example of typical capnographic wave analysis, using WinDaq software.

**Table 1:** Monitored variables during two 5 min tests within the open and sealed HEF

Variable	Label
**Identification and class variable**	

ID	Subject ID #
EXP	Test number (1-first or 2-second)
**EtCO_2 _wave parameters**	

POINTS	Number of CO_2 _measurements (20 samples/second) per one respiratory cycle
tot_len	Total time from STARTPOINT to ENDPOINT (seconds)
tot_AUC	Cross sectional area under the curve depicting detectable CO_2 _(mm Hg X seconds)
len2	Time from STARTPOINT to start of alveolar plateau (seconds)
slope2	Slope from STARTPOINT to start of alveolar plateau (mm Hg/second)
diff2	CO_2 _absolute change from STARTPOINT to start of alveolar plateau (mm Hg)
len3	Time from start to end of alveolar plateau (seconds)
slope3	Slope from start to end of alveolar plateau (mm Hg/second)
diff3	CO_2 _absolute change from start to end of alveolar plateau (mm Hg)
EtCO_2_	End tidal CO_2 _(maximum expired CO_2 _(mm Hg)
AUC3	Cross sectional area under the alveolar plateau (mm Hg X seconds)
**EtCO2 Wave Signal Statistics**	

MIN	Minimum (lowest) CO_2 _value from STARTPOINT to ENDPOINT
MAX	Maximum (highest) CO_2 _value from STARTPOINT to ENDPOINT
CORRMAX	Maximum (highest) CO_2 _value corrected for minimum (zero) value
MEAN	Mean CO_2 _value from STARTPOINT to ENDPOINT
MED	Median CO_2 _value from STARTPOINT to ENDPOINT
STDEV	Standard deviation of mean CO_2 _value from STARTPOINT to ENDPOINT
RMS	(true) Root mean square of CO_2 _values from STARTPOINT to ENDPOINT
**Minute Ventilation Variables**	

RR	Average respiratory rate (breaths/minute)
V_TE_	Average expiratory tidal volume (liters/BTPS)

From the list of variables in Table 1, *we selected *18 variables for statistical analyses. Nine describe aspects of the entire waveform and include the variables: tot_len, tot_AUC, EtCO_2_, CORRMAX, MIN, MEAN, STDEV, MED and RMS. Three describe phase II, from the start of the wave upstroke to the beginning of the alveolar plateau, and include len2, slope2 and absolute difference for slope2. Four describe phase III, the start to the end of the alveolar plateau, and include len3, slope3, diff3 and AUC3. Two were obtained from the minute ventilation test, immediately before and after the capnography test, reported as an average value, and include average RR and average V_TE_. Means, standard deviations, coefficients of variation (CV) and the number (N) of waveforms for each subject's test (in total EXP #1 = 60 waves and EXP #2 = 54 waves) were calculated for each of the 18 variables. The prefix M1 and M2 are the means for tests #1 and #2, S1 and S2 are the standard deviations and CV1 and CV2 the coefficients of variation (100 × standard deviation/mean). Data are reported for the four subjects, each of whom had two 5-minute capnography tests (8 tests in total). Among the 8 tests, the number of waveforms ranged from 8–19, depending on the subject's respiratory rate and acceptability of waveforms. Statistical analysis was performed using the SAS System for Windows v. 8.2 (SAS Institute Inc., Cary, NC., USA). The differences between the wave parameters, ventilatory parameters and signal statistics obtained within the open (EXP#1) and sealed (EXP#2) HEF, were evaluated using a paired t-test. A p-value < 0.05 was considered to indicate a statistically significant difference.

## Results

The results of the measurements for monitored variables are presented in Tables 2, 3, and 4. Summary data for EtCO_2 _parameters, comparing EXP#1 and EXP#2 for the four subjects are shown in Table 2. The two variables with the lowest CV were zero-corrected EtCO_2 _(CORRMAX), 3.89% for the first and 3.62% for the second measurement, and EtCO_2 _4.14% for the first and 4.48% for the second measurement, respectively. The phase II absolute difference (diff2) had a slightly higher CV, 6.48% and 6.52%. The eight other variables all had maximum CVs >10%, with the highest CV for the phase III slope (slope3), 52.57% and 57.96%. None of the mean values for these parameters measured with the HEF open and sealed showed any significant statistical difference. The p-values ranged from 0.16 for diff3 (phase III absolute difference) to 0.98 for len2 (length of the phase II).

**Table 2:** Descriptive statistics for capnographic wave parameters by exposure within open and sealed HEF and the paired t-test for EtCO_2 _wave parameters

EtCO_2 _Wave Parameters	EXP								Paired t-test (exp1-exp2)	
			
	1				2					
	
	N	Mean	Std	CV	N	Mean	Std	CV	t	p
Total length	60	3.53	0.63	17.9	54	3.39	0.69	20.32	0.62	0.58
Total area under the curve	60	84.62	16.85	19.92	54	78.68	16.89	21.46	0.85	0.46
phase II length	60	0.76	0.19	24.44	54	0.73	0.2	27.58	-0.02	0.98
phase II slope	60	44.46	10.19	22.92	54	45.41	10.38	22.86	0.49	0.66
phase II absolute difference	60	31.97	2.07	6.48	54	31.28	2.04	6.52	1.03	0.38
phase III length	60	1.63	0.39	24.02	54	1.52	0.41	26.96	0.81	0.48
phase III slope	60	3.15	1.66	52.57	54	3.49	2.02	57.96	-1.33	0.28
phase III area under the curve	60	59.40	13.44	22.63	54	54.86	14.36	26.18	0.64	0.57
phase III absolute difference	60	4.58	1.61	35.23	54	4.26	1.63	38.16	1.85	0.16
EtCO_2 _(signal maximum)	60	37.12	1.54	4.14	54	36.53	1.63	4.48	0.95	0.41
Zero-corrected EtCO_2_	60	36.84	1.43	3.89	54	35.83	1.3	3.62	1.29	0.29

**Table 3:** Descriptive statistics for ventilatory parameters by exposure within open and sealed HEF and the paired t-test for ventilatory parameters

Ventilatory Parameters	EXP								Paired t-test (exp1-exp2)	
			
	1				2					
	
	N	Mean	Std	CV	N	Mean	Std	CV	t	p
V_TE_	60	0.59	0.15	24.68	54	0.61	0.20	33.25	-0.33	0.76
RR	60	15.3	3.35	21.87	54	14.65	4.19	28.63	1.10	0.35

**Table 4:** Descriptive statistics for EtCO_2 _wave signal parameters by exposure within open and sealed HEF and paired t-test for EtCO_2 _signal parameters

EtCO_2 _Wave Signal Statistics	EXP								Paired t-test (exp1-exp2)	
			
	1				2					
	
	N	Mean	Std	CV	N	Mean	Std	CV	t	p|
MEAN	60	23.73	2.25	9.47	54	22.86	1.60	7.01	1.21	0.31
STDEV	60	13.85	1.14	8.21	54	13.81	0.69	5.01	0.16	0.88
MINIMUM	60	0.31	0.22	72.02	54	0.78	0.86	110.16	1.04	0.37
MEDIAN	60	30.60	3.81	12.47	54	29.55	3.59	12.15	0.59	0.60
RMS	60	27.49	1.75	6.37	54	26.67	1.45	5.43	1.21	0.31

Summary data for the ventilatory parameters, V_TE _and RR comparing EXP#1 and EXP#2 for the four subjects are shown in Table 3. The coefficients of variation for these two variables were above 10%, 21.87% for the CV of RR for the first measurement and 33.25% for CV of V_TE _for the second measurement. In addition, none of the mean values for these parameters was significantly different comparing the open versus sealed and operational HEF.

Descriptive statistics for EtCO_2 _signal variables measured within the open and sealed HEF (EXP#1 and EXP#2) are presented in Table 4. The coefficient of variation for RMS is smaller than the CV for MEAN, for both exposures, while the EtCO_2 _signal zero (MINIMUM) has the highest CV of all variables. The paired t-test for means showed no significant statistical difference between measurements within open and sealed HEF for any of these variables.

## Discussion

We added customized hardware and software components to interface with the HEF in order to utilize the advantages of capnography over the standard tests of lung function. We chose to use capnography in the HEF as we expect that capnography will be a useful tool for the detection of changes in ventilation during human exposure studies. Capnography, as a non-invasive, real-time physiologic measurement of adequacy of ventilation and perfusion, has found its role in a variety of medical disciplines including anesthesiology, critical care, emergency medicine, cardiology, pediatrics, neurology and respiratory care. Current capnographic hardware and software is designed for these aforementioned environments. As such, we did not find a commercially available, ready-to-go solution for the implementation of capnography in the experimental environment of human air pollution exposure studies. Our goal was to use relatively inexpensive and commercially available hardware components to acquire EtCO_2 _wave signals within sealed and operational HEF, but with acceptable signal quality. Our first choice for the capnograph was the Microcap^® ^CO_2 _portable bedside monitor, with nominal performance features adequate for our study's setup. However, due to the absence of an analog output, this monitor needed an additional digital-to-analog converter. We used one supplied as an option by the original manufacturer, designed to be used with this particular model of capnograph. Further processing of the four analog signals (EtCO_2 _wave, RR, EtCO_2 _wave signal validity and ground) utilized the standard solution (terminal board, expansion cable and MF 5500 AD card). Although the signal was less than 1.0 V, due to the low level of noise, we found that a single ended connection was adequate. We performed zero CO_2 _calibration of the capnograph with 100% O_2_, in order to measure the system signal noise for the overall hardware and software setup. The average noise level was 0.0052 ± 0.016 V and RMS was 0.0168 V. Signal-to-noise ratio (SNR) over the full scale of 100 mmHg was 34.59 dB. We found the WinView data acquisition software easy to install and setup using the MF 5500 AD board. The EtCO_2 _wave signal was saved as an ASCII file and imported into WinDaq Waveform Browser. This software was quite adequate for EtCO_2 _wave signal analysis.

An additional concern, related to the EtCO_2 _sampling line required a customized solution. We used two original sampling lines to make one custom line, which attaches on both sides of the custom-made needle port screwed into the plexiglas HEF window. Accordingly, we did not observe additional delay added to frequency response of the monitor. We considered the possibility that sampling through a longer line than originally designed and recommended by the manufacturer could also affect the performance of the capnography monitor. However, we did not observe that sampling in this way affected the reading of EtCO2 and RR on the capnograph itself. Mason et al. [36] showed that an experimental sampling line as long as 9.0 m was equally reliable as the standard 3.0 m long one, which agrees with our observation.

**Figure 4:**
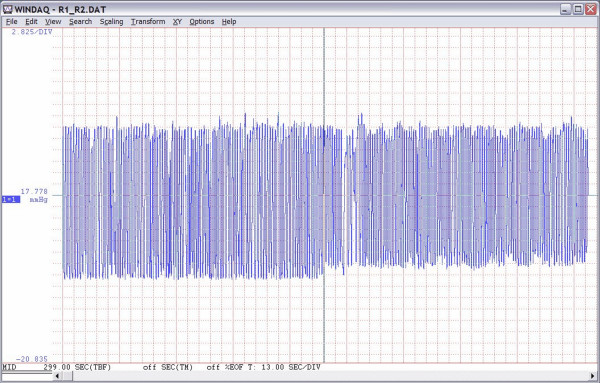
**Unexpected zero shift for one test out of eight**. Screenshot of WinDaq window showing trend capnography tracing for the EXP#1 (left side) and EXP#2 (right side) for the subject who had the particle delivery/CO_2 _removal mask incorrectly positioned during the EXP#2 that caused CO_2 _re-breathing.

Comparisons of the differences between capnographic measurements obtained under two different conditions, relevant to our design of human exposure studies (open and sealed HEF), did not yield any statistical significance. Yet, some differences existed in absolute values, but allocation of these differences among observed measures was to be expected. In the group of EtCO_2 _wave parameters, the variable with the lowest CV was the zero-corrected EtCO_2_, 3.89% and 3.62%, followed closely by EtCO_2_, 4.14% and 4.48%. Verschuren et al. [43] considered CV of the EtCO_2 _less than 5% as an indicator of steady-state status for their patients The EtCO_2 _is the main output value of the capnograph and correction of this value with the minimum-recorded value, for a particular wave, increased the accuracy of true EtCO_2 _reading. Among seven out of eight tests, we found that the range of recorded zero values for EtCO_2 _was between 0 and 0.55664 mmHg (1.42% of maximum-recorded EtCO_2 _of 39.167 mmHg). Surprisingly, we found that one subject's exposure (EXP#2 for that subject, with 15 acceptable analyzed waves) had recorded a zero range between 1.4274 to 2.8547 mmHg (7.29% of the maximum-recorded EtCO_2 _of 39.167 mmHg). Reviewing the lab log for this test, we found that 5 min after the enclosure was sealed, the recorded CO_2 _within the HEF was approximately 0.38%. This could have happened, because the particle delivery/CO2 removal mask either was not in place or correctly positioned, or in the case of the CO_2 _removal pump system malfunction. Thus, the maximum recoded zero for EtCO_2 _was approximately the background concentration of CO_2 _in the sealed HEF (749 mmHg × 0.0038 = 2.846 mmHg). Later, we were able to simulate this pattern of zero shift with the particle delivery/CO_2 _removal mask incorrectly positioned and this explained the unexpected CV of the MINIMUM variable for the EtCO_2 _signal. See Figure 4 for the EtCO_2 _tracing of that original test in trend format.

CV for the slopes of phase II and III, for both measurements, showed that these two variables are also the ones with the largest physiologic variability even in the steady state. Thus, they have the potential to be considered as variables capable of detecting a deviation from the normal pattern as well. Changes in RR, V_TE_, and ventilation maldistribution expressed as ventilation/perfusion (V/Q) mismatch, can result in changes of phase II and III of the EtCO_2 _wave [42]. For both, EXP#1 and EXP#2, RR and V_TE _were virtually identical, allowing us to have slopes of phase II and III clearly comparable between the two measurements. The CV for RR and V_TE _(range 21.87 to 33.25) were somewhat higher than reported by Verschuren at al. [43] for their patients. We believe that this might be possibly due to the psychological effect of the limited space in the HEF, which might affect the variability of the breathing pattern.

In the group of EtCO_2 _signal variables, we found that the CV for RMS was smaller than for the MEAN, because RMS has been calculated as the true RMS and better represents the "average" value of expired CO_2 _than the MEAN value obtained by the standard equation. The true RMS, in this case, better approximates mixed-expired CO_2 _(P_E_CO_2_) per one breath or over the time that the EtCO_2 _signal was "averaged". The P_E_CO_2 _and arterial partial pressure for CO_2 _(P_a_CO_2_) are used to calculate dead space to tidal volume ratio (V_D_/V_T_).

## Conclusion

We have described a customized, inexpensive and feasible hardware and software implementation of standard capnography, as an adjunct to our human exposure facility, which is used in air pollution studies. Our hardware and software setup for acquisition and analysis of EtCO_2 _wave signal was demonstrated as reliable and stable. We would have preferred that the selected capnograph monitor have an analog output, so we could avoid digital to analog signal conversion. The instrument was meant to be used in standard pre-hospital or hospital environments, therefore the manufacturer had not considered that option. However, extending the use of capnography into research labs like ours, where we carry out air pollution human exposure studies, has been long overlooked. Using capnography in the way we described, we may be able to detect and better characterize the effect of exposure to air pollution on cardiopulmonary system, particularly on lung ventilation and perfusion. We plan to use this technique in our on-going and subsequent controlled human exposure studies as a physiologic outcome measure.

## Authors' contributions

KZL initiated and designed the study, designed capnographic hardware & software setup, performed data analysis and interpretation, and drafted the manuscript. BU contributed to overall aspects of the study and specifically in statistical analysis, results interpretation and manuscript drafting. MF contributed in technical design modifications and conducted all experimental procedures and measurements. MEF contributed to study design and implementation with valuable clinical input and comments on physiologic aspects of capnography, while FS, the principal investigator, contributed valuable discussion and suggestions throughout this project. All authors read and approved the final manuscript.
